# A Survey of the Awareness and Educational Needs of Nurses in Nagasaki Prefecture Regarding Hereditary Breast and Ovarian Cancer

**DOI:** 10.1007/s13187-022-02132-4

**Published:** 2022-01-06

**Authors:** Megumi Matsumoto, Noriko Sasaki, Yayoi Tsukigawa, Ryota Otsubo, Hiroshi Yano, Takeshi Nagayasu

**Affiliations:** 1 Department of Surgical Oncology, Nagasaki University Graduate School of Biomedical Sciences, 1-7-1 Sakamoto, Nagasaki City, Nagasaki 852-8501 Japan; 2 Department of Reproductive Health, Institute of Biomedical Science, Nagasaki University, Nagasaki City, Nagasaki 852-8501 Japan; 3 Department of Nursing, Nagasaki University Hospital, Nagasaki City, Nagasaki 852-8501 Japan

**Keywords:** Educational program, Genetic screening, Hereditary breast and ovarian cancer, Oncology nurses

## Abstract

The aim of this study was to evaluate the knowledge and educational needs with regard to hereditary breast and ovarian cancer among nurses working in breast cancer care in the Nagasaki Prefecture. In breast cancer care, the identification of patients at risk for hereditary breast and ovarian cancer is necessary for the implementation of genetic testing and counseling. Nurses should be involved in this process, since they play a crucial role in the care of patients with breast cancer. However, the knowledge regarding hereditary breast and ovarian cancer among nurses working in oncology care in Japan has not been assessed. The design of this study is cross-sectional design. We distributed 597 surveys to nurses working in breast cancer care. The surveys assessed the nurses’ demographic data, their current knowledge and practices regarding cancer genetics and hereditary breast and ovarian cancer, and their attitude and preferences regarding learning about the condition. We received 317 valid replies. Nurses had limited knowledge about hereditary breast and ovarian cancer characteristics: 41.6% reported that they do not know about the condition, whereas less than 10% knew its characteristics. However, nurses were aware of hereditary breast and ovarian cancer significance and were willing to learn about it: 91% wished to learn about the condition, and 88.6% wanted to participate in study group meetings. Further, nurses’ preferences regarding educational programs were clarified. Overall, our results show that educational programs should be implemented to advance nurses’ knowledge of hereditary breast and ovarian cancer characteristics.

**Supplementary Information:**

The online version contains supplementary material available at 10.1007/s13187-022-02132-4.

## Introduction

Breast cancer is one of the most prevalent cancer types worldwide [1]. Hereditary breast and ovarian cancer (HBOC) syndrome is an inherited genetic condition associated with an increased risk of breast and ovarian cancer. Most cases of HBOC are caused by mutations in the breast cancer 1 (*BRCA1*) or breast cancer 2 (*BRCA2*) genes [2, 3]. The identification of patients with breast and/or ovarian cancer at high risk for HBOC is important to ensure appropriate genetic testing and counseling. Moreover, there is a high burden of need for genetic testing in patients at risk for HBOC [4]. To ensure adequate screening, genetic testing, and counseling for HBOC, healthcare providers should have sufficient knowledge in the field of cancer genetics and genomics [5]. However, many healthcare professionals do not receive sufficient training in genetics and genetic testing, which increases the risk of errors [6, 7].

Nurses play a critical role in the care of patients with breast cancer; therefore, they should be closely involved in screening for patients at high risk for HBOC. However, a survey conducted with Turkish oncology nurses showed that their knowledge of cancer genetics was only moderate [8, 9]. Similar findings were obtained in a Brazilian study, which found that the majority of nurses had not identified or referred a patient for specialized risk assessment due to a genetic risk [10]. Only a few studies have assessed the knowledge of healthcare providers in Japan regarding HBOC screening, testing, and counseling, and none focused on nurses. A previous investigation found that although there was an awareness of the significance of HBOC testing among Japanese healthcare providers, it was generally not sufficiently implemented in practice, and there was a need to further educate nurses. However, that study primarily assessed the knowledge and educational needs of clinical geneticists, other physicians, and genetic counselors, whereas only 2.4% of the respondents were nurses [11]. Moreover, in a study limited to physicians, genetics training and continuing medical education were associated with better knowledge of HBOC risk assessment [12].

The authors’ institution has the capacity to construct a medical care system for HBOC primary screening and identification, risk assessment, genetic counseling, genetic testing, surveillance, and management of risk-reducing surgery. However, even though it is the only institution capable of providing HBOC care in the prefecture, there have been few cases of genetic counseling (47 cases between 2014 and 2016). Hospitals in the prefecture, including this institution, have not yet succeeded in placing appropriate cases on the care flow as HBOC cases.

In addition to their knowledge of HBOC, the educational needs of nurses working in breast cancer care should also be assessed. The necessity for further genetic education has previously been described among nurses in other settings [13]. However, the knowledge and specific educational needs of nurses working in cancer care in Japan have not been analyzed.

The aim of this study was to gain insight into the level of understanding, interest, and educational needs of nurses regarding HBOC by conducting a survey among nurses working in the field of breast cancer care in Nagasaki Prefecture.

## Materials and Methods

### Study Design and Participants

Nurses working in the field of breast cancer care at 23 institutions in Nagasaki Prefecture were contacted for participation in this descriptive study. The study was approved by the Ethics Committee of the Nagasaki University Hospital. It was conducted between June 1, 2016 and November 30, 2016. A questionnaire was distributed via physicians at each institution, and answers were collected from each respondent by mail. By mailing in their replies, the respondents were deemed to have provided consent. The total number of distributed questionnaires was 597, while the number of valid responses was 317 (53.1%).

### Study Survey

The survey was developed by the authors of this study and included 14 questions. The first part of the survey (questions 1–4) gathered information about the participants’ age, sex, professional qualifications, and current work situation. The second part (questions 5–10) inquired about nurses’ current knowledge and practices regarding cancer genetics and HBOC. These questions clarified whether the nurses had been involved in breast cancer care and for how long, whether and to what extent they had collected the family history of patients with breast cancer, whether and to what extent they were aware of HBOC, and whether they had received a question or consultation request regarding genetics by a patient with breast cancer. The third part of the survey (questions 11–14) assessed whether nurses had had the opportunity to learn about HBOC, whether they wanted to learn about it, what their motivation for learning was, and what their preferred learning format was.

### Statistical Analysis

The multiple-choice questions included in the survey were assessed with descriptive statistics, whereas the open-ended questions were evaluated using content analysis.

## Results

### Participants’ Characteristics

The majority of the participants were women (97%). More nurses worked in a ward (70%) than in an outpatient setting (27%). The most prevalent duration of experience working with breast care was 1–5 years (47% of nurses working in wards and 59% of nurses working in outpatient units) (Supplementary Table S1).

### Nurses’ Practices with Respect to Family History Assessment

Slightly more than half of the nurses (52%) participating in the study reported that they were aware of the family history of patients with cancer. Moreover, 63% responded that they asked patients with cancer about their family history. However, only 23% of nurses who recorded a family history also asked about the family history of extended family members up to third-degree relatives (Table 1).

**Table 1:** Current practices of the nurses with respect to family history

Questions	Possible answers	Rates
Do you ask about the family history of patients? (*n* = 317)	Yes	63%
	No	36%
	Unknown	1%
Do you ask about the family history of the extended family members up to the third-degree relatives? (If the answer to the previous question is yes, *n* = 199)	Yes	23%
	No	75%
	Unknown	2%
When you see a cancer patient, are you aware of their family history? (*n* = 317)	I am aware	52%
	I am not aware	48%

### Nurses’ Knowledge and Practices with Respect to HBOC and Its Screening

**Fig. 1:**
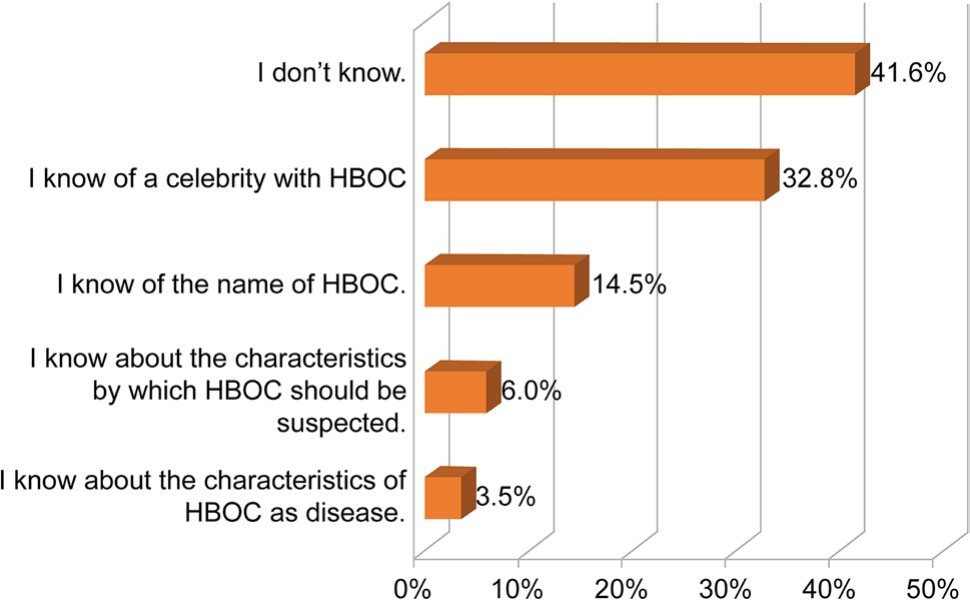
The degree of nurses’ knowledge regarding hereditary breast and ovarian cancer

When the nurses were asked whether they knew about HBOC, 41.6% reported that they did not know about it, whereas 58.4% reported that they did. However, fewer than 10% of the respondents knew about the characteristics of HBOC or the characteristics leading to suspicion of HBOC (Fig. 1).

Open-ended questions were also asked about the knowledge and practices of nurses with respect to questions or consultation requests concerning genetics by breast cancer patients (Supplementary Table S2). The most frequently asked question was “Can my close relatives (especially my daughter) inherit the cancer from me?” The most common answer to this question was “I recommended a self-examination and a breast cancer screening test.”

### Attitudes of Nurses Regarding Learning About HBOC

Ninety-one percent of the participants in the survey were willing to learn about HBOC; furthermore, 88.6% of the respondents wished to participate in HBOC study group meetings. However, before the survey, the majority of respondents had had the opportunity to learn only about breast cancer (62%) but not about heritability (3%). The most important reasons for wishing to learn were wanting to utilize this knowledge to provide information when being questioned (86.6%) or to implement it in nursing (53.4%) (Table 2).

**Table 2:** Nurses’ attitudes regarding learning about inheritance and hereditary breast and ovarian cancer (HBOC)

Questions	Answers	Rates
Have you had an opportunity to learn about cancer and inheritance? (*n* = 317)	I only had an opportunity to learn about breast cancer	62%
	I only had an opportunity to learn about inheritance	3%
	I had an opportunity to learn about both	9%
	I have had neither learning opportunity	26%
Do you wish to learn about HBOC? (*n* = 317)	Yes	91%
	No	8%
	Unknown	1%
Reasons for wishing to learn (*n* = 281)	I would like to utilize what I learn to provide information when being questioned	86.6%
	I would like to implement what I learn in nursing	53.4%
	It is useful for me and people close to me, such as relatives	31.4%
	It is a hot topic these days	15.2%
Reasons for not wishing to learn (*n* = 26)	I do not feel a particular need to learn	11
	I am not interested	9
	There is no established system yet, etc	6
Topics nurses want to learn about (*n* = 281)	HBOC pathology (incidence rates of cancer, inheritance, etc.)	76.0%
	Basic information about inheritance	68.5%
	The reality of HBOC care (identifying patients, asking about the family history, etc.)	61.5%
	Genetic testing	47.6%
	Genetic counseling	33.1%
Do you wish to participate in a study group meeting on HBOC? (*n* = 317)	I wish to participate	88.6%
	I do not wish to participate	7.9%
	Unknown	3.5%
How long should the study group meeting sessions be?	Less than 30 min	12.5%
	1 h	62.1%
	2 h	12.8%
	Over 2 h	1%
	Unknown	11.6%
Wishes for the study group meetings (open-ended)	Short duration/multiple times/serialization	
	e-Learning/on-demand/live	
	Inclusion of physicians	
	Provision of documents	
	Q&A of frequently asked questions	
	Held on weekends/in the evenings after work	
How do you check on what you do not know during nursing work? (*n* = 317)	Internet	85.5%
	Journals (nursing journals, academic journals, etc.)	70.3%
	Questioning an acquaintance	36.0%

The topics nurses wanted to learn about included the pathology of HBOC (76%), basic information about inheritance (68.5%), the reality of HBOC care (61.5%), genetic testing (47.6%), and genetic counseling (33.1%). The majority of nurses were interested in hour-long study sessions. The internet (85.5%) and journals (70.3%) were the most frequently used sources for learning (Table 2).

## Discussion

The current study found that knowledge regarding HBOC among Japanese nurses working in the field of breast cancer care is limited, whereas their interest in learning more about it is pronounced.

In this study, the nurses had limited knowledge of HBOC inheritance. These findings agree with previous publications, which showed limited knowledge of hereditary breast cancer/HBOC among nurses in Brazil and Turkey, especially with respect to genetic counseling [9, 10]. Our study was based on self-assessment of HBOC knowledge among nurses. However, the utilization of objective assessment tools may provide more reliable information. Such tools should focus on examining nurses’ knowledge of cancer genetics and HBOC. A range of factors should be assessed, including knowledge of the risk factors, epidemiology, inheritance patterns, screening, diagnosis, and treatment for HBOC. Similar questionnaires have been developed and have been successfully implemented to assess the knowledge of nurses regarding hereditary breast cancer [9, 10]. Moreover, future studies should compare HBOC awareness between nurses from cancer centers that have and have not implemented HBOC educational programs.

The nurses were aware of the significance of HBOC awareness and screening. A previous study showed that involving nurses in screening for HBOC could help identify patients who required further testing [14]. Moreover, nurses’ knowledge regarding pharmacogenetics and pharmacogenomics in general may improve patient outcomes [15]. Therefore, the results of the present and previous studies indicate that genetics and genomics knowledge is critical for oncology nurses [16].

The current study found that nurses are motivated to learn about HBOC characteristics, mainly to answer patients’ questions and to implement the newly acquired knowledge in nursing. Previous studies also found that the majority of nurses are motivated to continue their education in the field of cancer genetics [9, 10].

The development of web-based educational interventions in the fields of genetics and genomics is an area of active research [17]. Standards for the core competencies of healthcare professionals in the field of genomic medicine have been established to serve as a basis for the development of educational programs [18]. These standards relate to the domains of knowledge, attitudes, and abilities [5]. Educational genetics interventions, including interactive workshops and PowerPoint modules, have also been developed and have shown to be effective in educating primary healthcare providers [19]. In this survey, the nurses reported that they used the internet as a primary source of learning. In Nagasaki Prefecture, there are many minor islands, and interactive study group meetings via e-learning and internet are being considered by our team and institution, including in remote areas.

Promoting nurses’ knowledge of cancer genetics will also have beneficial effects on patient education. For example, education for breast self-examination of women and *BRCA1* or *BRCA2* mutations has been proven to be feasible [20].

This study has several limitations. First, it used self-report measures to assess nurses’ awareness of HBOC. These findings should be confirmed through more objective assessments. Second, it did not directly evaluate the effect of nurses’ knowledge regarding HBOC on the effectiveness of patient screening and the identification of patients at high risk for HBOC. Third, even though it assessed nurses’ preferences regarding the development of an educational program, further studies should determine the optimal design of the program.

## Conclusion

The results of this awareness survey among nurses working in breast cancer care institutions in Nagasaki Prefecture indicate that most nurses do not know about the disease characteristics and inheritance patterns of HBOC but are eager to learn about them. Therefore, further focused learning opportunities should be provided to nurses working in breast cancer care.

## Supplementary Information

Below is the link to the electronic supplementary material.

Supplementary file1 (DOCX 16 KB)

## Data Availability

All data generated or analyzed during this study are included in this published article (and its supplementary information files).
