# Supplementary material for: A Survey of the Awareness and Educational Needs of Nurses in Nagasaki Prefecture Regarding Hereditary Breast and Ovarian Cancer
Source: J Cancer Educ. 2022 Jan 6;38(2):406–11. doi: 10.1007/s13187-022-02132-4 (PMC10102085; doi:10.1007/s13187-022-02132-4)
Supplement: Supplementary file 1 — Supplementary file1 (DOCX 16 KB) [file 13187_2022_2132_MOESM1_ESM.docx]

**A SURVEY OF THE AWARENESS AND EDUCATIONAL NEEDS OF NURSES IN NAGASAKI PREFECTURE** **REGARDING HEREDITARY BREAST AND OVARIAN CANCER**

**Supplementary Table S1.** Participants’ characteristics

| **Variables** |  | **Rates** |
| --- | --- | --- |
| Sex | Female  Male | 97%  3% |
| Age group | 20–29 years  30–39 years  40–49 years  50–59 years  Other/unknown | 29%  22%  28%  19%  2% |
| Workplace | Ward  Outpatient unit  Other/unknown | 70%  27%  3% |
| Years of experience with breast care for nurses working in wards | < 1 year  1–5 years  6–10 years  ≥ 11 years  Uncertain | 8%  47%  18%  3%  24% |
| Years of experience with breast care for nurses working in outpatient units | < 1 year  1–5 years  6–10 years  ≥ 11 years | 8%  59%  30%  3% |
| Professional certification | No additional certifications  Certified nurse specialist  Certified nurse  Midwife  Public health nurse  Care manager  Other  Multiple qualifications | 282  2  16  4  4  4  8  2 |

**Supplementary Table S2.** Open-ended questions revealing the knowledge and practices of nurses with respect to genetic questions or consultation requests from patients with breast cancer

| **Questions** | **Answers** |
| --- | --- |
| Is breast cancer hereditary?  (n=5) | I asked a physician to respond to the question (n=2).  I explained that there are inherited and non-inherited cancers (n =2).  I recommended a screening test (n =1). |
| Is my breast cancer caused by inheritance?  (n=4) | It is not necessarily 100% related to inheritance (n=1).  There is a possibility of it being inherited (n=1).  Inheritance could be the cause if your mother or sister had breast cancer (n=1).  I do not remember (n=1). |
| Can my close relatives (especially my daughter) inherit the cancer from me?  (n=8) | I asked a physician to respond to the question (n=2).  I recommended a self-examination and a breast cancer screening test (n =5).  I referred the patient to a University Hospital (n=1). |
